# Severe Atopic Dermatitis With Increasing Daytime Sleepiness Following Nemolizumab Administration: A Case Report

**DOI:** 10.7759/cureus.78214

**Published:** 2025-01-29

**Authors:** Kenta Horimukai, Misako Kinoshita, Noriko Takahata

**Affiliations:** 1 Department of Pediatrics, Jikei University Katsushika Medical Center, Katsushika, JPN

**Keywords:** atopic dermatitis, interleukin-31, nemolizumab, pruritus, quality of life (qol), sleep wake disorders

## Abstract

Atopic dermatitis (AD) often impairs daytime activities and quality of life (QOL) owing to intense pruritus, which disrupts sleep. Nemolizumab, an interleukin (IL)-31 receptor α inhibitor, has emerged as a promising treatment that can alleviate itching and enhance both nighttime rest and daytime functioning. We report the case of a 16-year-old boy with severe AD that was refractory to multiple therapies, including topical steroids, immunomodulators, topical Janus kinase (JAK) and phosphodiesterase 4 (PDE4) inhibitors, moisturizers, and oral antihistamines. Subcutaneous administration of nemolizumab was initiated, resulting in significant improvements in the Eczema Area and Severity Index (EASI), Patient-Oriented Eczema Measure (POEM), and Atopic Dermatitis Control Tool (ADCT) scores. However, he exhibited a marked increase in the Epworth Sleepiness Scale (ESS) the day after each injection, accompanied by pronounced daytime drowsiness lasting one to two weeks. Upon discontinuation of nemolizumab at the patient’s request, daytime sleepiness resolved. Although nemolizumab is expected to reduce pruritus and improve daytime alertness, our patient exhibited paradoxical post-injection hypersomnolence. Possible contributors include shifts in the circadian rhythm or central nervous system effects related to IL-31 inhibition, although the precise mechanism is yet to be established. Therefore, careful monitoring of both dermatologic outcomes and daytime vigilance is essential. Clinicians should be aware of potential post-injection sleepiness when administering nemolizumab, given that it is an otherwise effective treatment for severe AD.

## Introduction

Atopic dermatitis (AD) is a chronic inflammatory skin condition characterized by intense pruritus, which disrupts sleep and impairs daytime functioning, thereby reducing the quality of life (QOL) [1]. Interleukin (IL)-31, recognized as a cytokine that induces pruritus, is produced by T helper type 2 (Th2) cells and exerts its effects on sensory neurons through the IL-31RA/OSMRβ receptor complex. In TRPV1-positive dorsal root ganglion (DRG) neurons, it promotes STAT3-dependent branching and elongation of neuronal processes. Moreover, by increasing the expression of GRP and suppressing GABA-B1 receptor expression in the dorsal horn of the spinal cord, IL-31 leads to a state of central sensitization [2].

Nemolizumab, a biologic agent targeting the IL-31 receptor α, was developed to alleviate refractory pruritus and improve both nighttime sleep and daytime functioning of patients with AD [3]. Large-scale clinical trials, including the ARCADIA studies, demonstrated that nemolizumab significantly reduces itching and improves sleep quality, thus bolstering daytime alertness and overall QOL [4]. Nonetheless, we encountered a 16-year-old boy with severe AD who developed pronounced daytime sleepiness starting the day after each dose despite notable improvements in pruritus and skin lesions following nemolizumab treatment.

While the expected clinical course is “pruritus reduction → improved nocturnal sleep → enhanced daytime alertness,” this patient diverged from that pattern, prompting consideration of IL-31 signalling and sleep-wake regulation. It has been reported that 7.79% of children aged 6 to 18 years (mean age 14.18 ± 16.67 years) received omalizumab - a biologic agent in widespread clinical use - experienced drowsiness as an adverse event [5]. Although nemolizumab may likewise pose a potential risk of somnolence as an adverse effect, there is very little prior research examining this issue. Here, we describe this case and discuss the possible pathophysiological mechanisms underlying the observed increase in daytime sleepiness. Written informed consent was obtained from the patient and his family prior to publication.

## Case presentation

A 16-year-old boy had severe AD since age eight, with frequent flare-ups despite the use of topical steroids, immunomodulators, topical Janus kinase (JAK) and phosphodiesterase 4 (PDE4) inhibitors, moisturizers, and oral antihistamines. He also had allergic rhinitis (treated with mometasone nasal spray), bronchial asthma (treated with montelukast), and a partial egg avoidance diet for food allergies. Persistent pruritus interfered with his school baseball practice. Baseline laboratory data showed markedly elevated total IgE (53,985 IU/mL) and thymus and activation-regulated chemokine (TARC) (2,621 pg/mL) levels. His white blood cell count was 26,100/μL with 13.1% eosinophils, indicating a high inflammatory burden. He was 163.9 cm tall (-0.46 SD), weighed 49.8 kg (-0.68 SD), and had a BMI of 18.5, indicating the absence of obesity. There was no evidence of micrognathia. Although nasal obstruction had been reported prior to nemolizumab therapy, the otoscopic evaluation revealed only mild swelling of the inferior turbinate mucosa at baseline. The following sections summarize his clinical course after initiating nemolizumab.

Baseline (week 0)

Despite ongoing treatment, his AD remained poorly controlled: EASI 11.5, POEM 13, ADCT 12, and pruritus Numerical Rating Scale (NRS) 5. His Epworth Sleepiness Scale (ESS) was 5.

First nemolizumab injection (week 0)

Subcutaneous nemolizumab (60 mg every four weeks) was started. By day 1 post-injection, his ESS rose from 5 to 11, accompanied by marked afternoon drowsiness. Although his EASI increased slightly (11.5 → 12.1), by week 1 his skin condition improved (POEM 13 → 1, ADCT 12 → 4, pruritus NRS 5 → 2), whereas ESS remained elevated at 9.

Second injection (week 4)

Following the second dose, daytime pruritus remained low (NRS 2). However, ESS again increased (8 → 10) by the next day. By week 8, his EASI had improved to 5.25, and ESS briefly declined to 6.

Third injection (week 8)

After the third injection, ESS surged to 11, leading to near “microsleeps” during class.

Decision to discontinue (week 13)

By week 13, his ESS returned to 5, and AD remained stable, but repeated episodes of post-injection somnolence prompted the discontinuation of nemolizumab. His caregiver also reported increased drowsiness during treatment, which resolved once the medication was stopped. Up to week 13, there was no worsening of inferior turbinate mucosal thickening.

Follow-up (week 23)

Ten weeks after nemolizumab was discontinued, his EASI increased slightly (6.9 → 10.25). Nevertheless, pruritus remained stable, and his ESS was 5 without notable daytime sleepiness.

**Figure 1:**
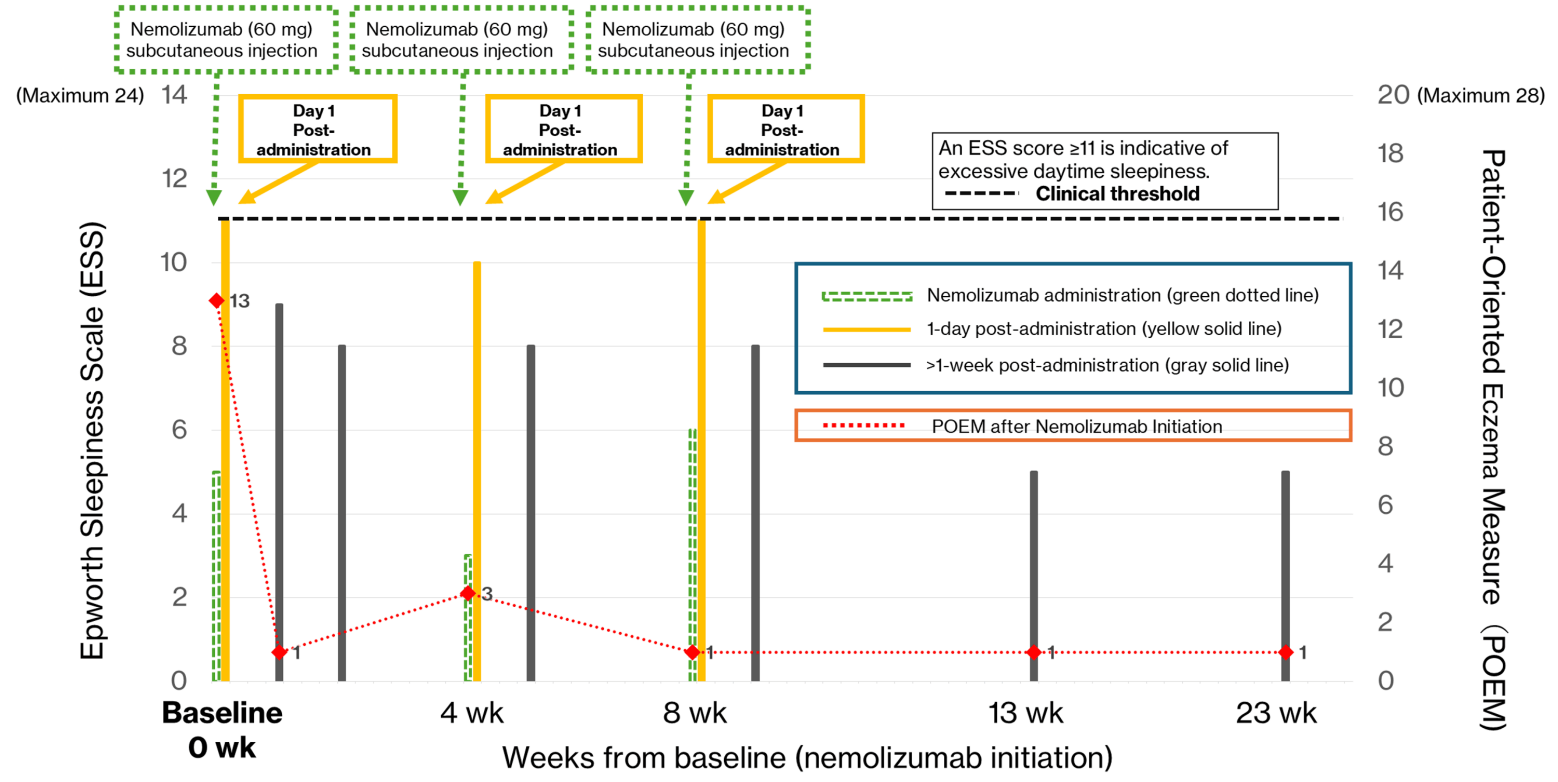
Changes in the Epworth Sleepiness Scale (ESS) and Patient-Oriented Eczema Measure (POEM) Over Time Following Nemolizumab Initiation in a Single Patient 1. Baseline is defined as week 0 (before nemolizumab initiation). 2. The ESS is scored from 0 to 24 points. For clarity, only values up to 14 are shown on the left axis. The clinical threshold for excessive daytime sleepiness (ESS ≥11) is indicated by the black dashed line. 3. POEM is plotted on the right axis, with a maximum score of 28. 4. Each symbol and line represents the same patient’s longitudinal data.

More detailed clinical measurements are provided in Table 1. Figure 1 illustrates the time course of ESS, EASI, and POEM throughout therapy. Footnotes to Table 1 contain additional information on each parameter.

**Table 1:** Clinical Assessments Over Time in a Patient Receiving Nemolizumab Footnotes: 1. Weeks are indicated relative to the first dose of nemolizumab (week 0). 2. Eczema Area and Severity Index (EASI): A validated tool for atopic dermatitis severity. EASI is scored from 0 to 72. 3. Patient-Oriented Eczema Measure (POEM): A patient-reported instrument used to assess atopic dermatitis severity, scored from 0 to 28. 4. Atopic Dermatitis Control Tool (ADCT): A brief survey measuring disease control from the patient’s perspective. The total score ranges from 0 to 24. 5. Pruritus Numerical Rating Scale (NRS): A measure of itch intensity ranging from 0 (no itch) to 10 (worst imaginable). 6. Epworth Sleepiness Scale (ESS): A self-reported measure of daytime sleepiness ranging from 0 to 24. 7. TARC, SCCA2, LDH: Laboratory biomarkers detailed in the main text. They serve as common inflammatory markers that can reflect the severity of atopic dermatitis. 8. Daytime and nocturnal pruritus scores are evaluated on a 0-4 scale unless otherwise noted. 9. “Week X + 1 day” indicates the day following the specified week relative to the initial nemolizumab dose. SCCA2: squamous cell carcinoma antigen 2; TARC: thymus and activation-regulated chemokine; TEWL: transepidermal water loss Notes: 1. Values for “–” indicate no available data at that time point. 2. Some columns note “Nemolizumab initiation” or “Nemolizumab administration” to clarify dosing time points. 3. Bold text indicates marked sleepiness, which is defined as a total ESS score of 11 or higher, with each item scoring 2 or above.

	Week 6	Week 0	Week 0 + 1 day	Week 1	Week 2	Week 4	Week 4 + 1 day	Week 5	Week 8	Week 8 + 1 day	Week 9	Week 13	Week 23
	–	Nemolizumab initiation	–	–	–	Nemolizumab administration	–	–	Nemolizumab administration	–	–	–	–
Total ESS	–	5	11	9	8	3	10	8	6	11	8	5	5
Sitting and reading	–	1	1	1	1	1	1	1	1	1	1	1	1
Watching television	–	0	1	1	1	0	1	1	0	1	1	0	0
Sitting in a public inactive place (theater or meeting)	–	0	1	1	1	0	1	1	1	1	1	1	0
Riding in a car for one hour without a break (as a passenger)	–	1	2	2	2	1	2	2	2	3	2	1	1
Lying down in the afternoon when circumstances permit	–	2	3	2	2	1	3	1	1	3	2	1	1
Sitting and talking to someone	–	0	1	0	0	0	1	0	0	1	0	0	0
Sitting quietly after lunch, without alcohol	–	0	1	1	1	0	0	1	0	0	0	0	1
Stopped in traffic for a few minutes	–	1	1	1	0	0	1	1	1	1	1	1	1
EASI	10.1	11.5	–	12.1	–	10.5	–	–	5.25	–	–	6.9	10.25
POEM	–	13	–	1	–	3	–	–	1	–	–	1	1
ADCT	–	12	–	4	–	2	–	–	1	–	–	1	1
Pruritus NRS	6	5	–	2	–	3	–	–	4	–	–	3	2
Daytime pruritus score	3	3	–	–	–	2	–	–	2	–	–	2	2
Nocturnal pruritus score	4	3	–	–	–	2	–	–	2	–	–	2	2
TEWL (inner side of left forearm)	–	15.9	–	–	–	26.4	–	–	12.1	–	–	10.5	12.4
TEWL (outer side of right forearm)	–	18.2	–	–	–	15.7	–	–	16.5	–	–	11.5	13.3
TEWL (outer side of left lower leg)	–	9.3	–	–	–	27.4	–	–	6.8	–	–	7.3	11.5
TARC	–	2621	–	–	–	–	–	–	–	–	–	–	–
SCCA2	–	11.1	–	–	–	–	–	–	–	–	–	–	–
LDH	–	221	–	–	–	–	–	–	–	–	–	–	–

## Discussion

Despite substantial improvements in pruritus and skin scores (EASI, POEM, and ADCT), the patient repeatedly reported pronounced daytime sleepiness, starting on the day after each dose. In this study, the ESS was employed to evaluate the patient’s level of daytime somnolence. In 1991, Johns developed the ESS, an internationally recognized eight-item measure used to subjectively assess excessive daytime sleepiness [6]. The Japanese version of this scale, known as the Japanese Version of the Epworth Sleepiness Scale (JESS), was created under the supervision of the Japanese Respiratory Society and has been reported to have sufficient validity [7].

In AD, nocturnal pruritus often disturbs sleep and its reduction usually enhances alertness. Notably, this patient’s ESS score rose consistently despite significant relief from pruritus. While prior trials of nemolizumab have noted better sleep quality, few have highlighted an increase in daytime drowsiness. However, a phase III study suggested that sleep-related problems may persist beyond week 16 [8], suggesting that such effects may be overlooked.

One explanation is the rapid circadian realignment after an abrupt improvement in fragmented sleep, causing transient dysregulation of the sleep-wake cycle [9,10]. Alternatively, IL-31 inhibition may indirectly affect the histaminergic and orexinergic neurons, which are essential for arousal [11]. However, no definitive mechanism has yet been established. As previously described, omalizumab has been associated with somnolence at a frequency similar to that of a headache, making it one of the more commonly reported adverse events [12,13]. However, the precise mechanism underlying omalizumab-induced somnolence remains unknown. Somnolence has also been reported with dupilumab, which is widely used worldwide. According to the World Health Organization pharmacovigilance database (VigiBase), out of 94,065 adverse events for dupilumab, 1,294 (1.4%) were classified as sleep-related, including insomnia and somnolence [14]. Although somnolence accounted for only 0.09% (81/94,065) of all events, this illustrates that drowsiness is a recognized but rare adverse effect of certain biologics. Mechanisms likely vary among agents; therefore, clinicians should be aware of such presentations in clinical practice.

A limitation of this case is that daytime somnolence was primarily assessed by the ESS, raising the potential for recall bias from partial retrospective data. Even so, previous reports that implicated somnolence as an adverse reaction to biologics other than nemolizumab also relied exclusively on patient-reported outcomes, leaving objective measures underexamined and the mechanism unresolved. In other words, the issue of biologic-induced somnolence remains insufficiently explored. Therefore, when prescribing these agents, it is vital to incorporate sleep evaluations - such as periodic ESS assessments - into standard clinical practice. Furthermore, objective techniques, including polysomnography and actigraphy, may provide robust data [15]. Collecting such evidence could facilitate the integration of routine sleep assessments into post-marketing surveillance.

Accordingly, larger or multicenter studies are needed to determine the optimal dosing intervals for nemolizumab and address daytime drowsiness. Further research on the impact of IL-31 inhibition on the central nervous system is also warranted.

In summary, nemolizumab effectively mitigates treatment-resistant AD-related pruritus and usually improves sleep quality, yet paradoxically provokes pronounced sleepiness in some instances. If somnolence is suspected during treatment with any biological agent, strategies such as extending the dosing interval may be considered, and we anticipate the development of clearer management guidelines. Moreover, periodic monitoring of alertness using the ESS or other objective sleep parameters is recommended to guide therapeutic decisions and ensure patient safety.

## Conclusions

Nemolizumab substantially improved severe AD in this patient but consistently triggered heightened daytime sleepiness following each dose. Possible mechanisms include circadian instability or altered neural regulation linked to IL-31 inhibition; however, the precise pathway remains unclear. Clinicians should closely monitor patients for excessive daytime somnolence and adjust treatment plans accordingly, and they may also contribute relevant data to future post-marketing surveillance and large-scale studies. Additional case reports and objective sleep evaluations are crucial for clarifying the underlying pathophysiology.
